# Supplementary figures and images for: From Lucy to Kadanuumuu: balanced analyses of Australopithecus afarensis assemblages confirm only moderate skeletal dimorphism
Source: PeerJ. 2015 Apr 28;3:e925. doi: 10.7717/peerj.925 (PMC4419524; doi:10.7717/peerj.925)

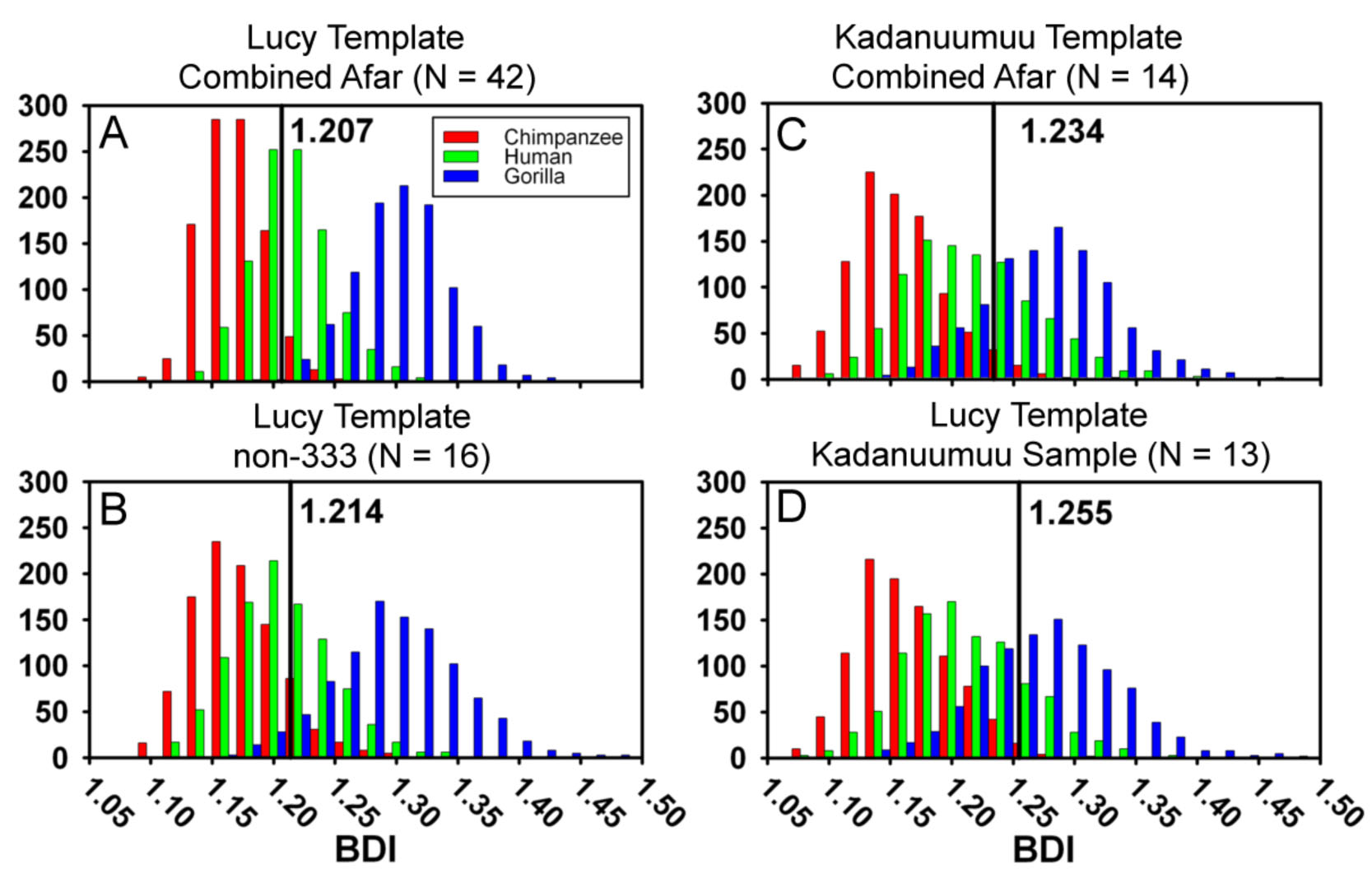

Supplement: Figure S1 — The vertical line and number indicate the dimorphism value (BDI) for the respective Au. afarensis sample. The BDI produces essentially similar results as the CV depicted in Fig. 3. [file peerj-03-925-s001.jpg]
