# Supplementary material for: From Lucy to Kadanuumuu: balanced analyses of Australopithecus afarensis assemblages confirm only moderate skeletal dimorphism
Source: PeerJ. 2015 Apr 28;3:e925. doi: 10.7717/peerj.925 (PMC4419524; doi:10.7717/peerj.925)
Supplement: Table S1 — Bold indicates the values used for the simulations limiting A.L. 128/129 and Lucy contribution to a single metric. The values for Lucy increased by 130% (see text) are shown in italics. [file peerj-03-925-s002.docx]

**Supplemental Table 1.** The Lucy (A.L. 288-1) template sample used to calculate Geometric Mean Method dimorphism. Bold indicates the values used for the simulations limiting A.L. 128/129 and Lucy contribution to a single metric. The values for Lucy increased by 130% (see text) are shown in italics.

**Specimen HHD OLCB CAPD RHD ULB FHD TRCD FNKH GSTB CNDC PXTB DSTB FIBD TAL**

A.L. 128/129 - - - - - - 26.6 - 21.7 26.8 **51** - - -

A.L. 137-48A - 37.1 - - - - - - - - - - - -

A.L. 137-50 - 43.5 - - - - - - - - - - - -

A.L. 152-2 - - - - - 33.1 - - - - - - - -

A.L. 288-1 26.5 32.5 14.4 15.1 14.2 **28.6** 26.2 21.8 23.3 27.5 50.2 17.2 18.9 37.4

*(130% A.L. 288-1*) (*34.5*) (*42.3*) (*18.7*) (*19.6*) (*18.5*) (*37.2*) (*34.0*) (*28.3*) (*30.3*) (*35.8*) (*65.3*) (*22.4*) (*24.6*) (*48.6*)

A.L. 211-1 - - - - - - 33.3 - - - - - - -

A.L. 223-23 - 40.1 - - - - - - - - - - -

A.L. 322-1 - 31.7 16.2 - - - - - - - - - - -

A.L. 330-6 - - - - - - - - - - 65.7 - - -

A.L. 333-3 - - - - - 40.9 - 29.1 - - - - - -

A.L. 333-4 - - - - - - - - 28.7 - - - - -

A.L. 333-6 - - - - - - - - - - - 22.4 - -

A.L. 333-7 - - - - - - - - - - - 25.8 - -

A.L. 333-9A - - - - - - - - - - - - 28.3 -

A.L. 333-9B - - - - - - - - - - - - 25.7 -

A.L. 333-29 - 37.7 - - - - - - - - - - - -

A.L. 333-42 - - - - - - - - - 35.3 - - - -

A.L. 333-85 - - - - - - - - - - - - 26.8 -

A.L. 333-95 - - - - - - 32.3 - - - - - - -

A.L. 333-96 - - - - - - - - - - - 23.1 - -

A.L. 333-107 36.5 - - - - - - - - - - - - -

A.L. 333-117 - - - - - - - 29.5 - - - - - -

A.L. 333-123 - - - - - - - 25.1* - - - - - -

A.L. 333-140 - - - - - - - - 24.6* - - - - -

A.L. 333-142 - - - - - - - 22.9* - - - - - -

A.L. 333-147 - - - - - - - - - - - - - 47.1

A.L. 333w-22 - - 19.9 - - - - - - - - - - -

A.L. 333w-31 - 39.0 - - - - - - - - - - - -

A.L. 333w-36 - - - - 14.8 - - - - - - - - -

A.L. 333w-37 - - - - - - - - - - - - 25 -

**Supplemental Table 1.** Continued.

**Specimen HHD HOCB CAPD RHD ULB FHD TRCD FNKH GSTB CNDC PXTB DSTB FIBD TAL**

A.L. 333w-56 - - - - - - - - 27.4 - - - - -

A.L. 333x-5 - - - - 18.4 - - - - - - - - -

A.L. 333x-14 - - - 23.4 - - - - - - - - - -

A.L. 333x-15 - - - 23.5 - - - - - - - - - -

A.L. 333x-26 - - - - - - - - - 37.0 - - - -

A.L. 438-1a - - - - 20.3 - - - - - - - - -

A.L. 444-14 - - 18.7* - - - - - - - - - - -

A.L. 545-3 - - - - - - - - - - - 19.2* - -

A.L. 827 - - - - - 38.1 - - - - - - - -

KSD-VP-1-1 - - - - - - - - - - - 26.8 - -

MAK-V/P-1/1 - - - - - - 31.5 23.8 - - - - - -

MAK-V/P-1/3 - 43.0 - - - - - - - - - - - -

* Data from (Harmon, 2006, *J Hum Evol* 51, 217-227) or (Ward et al., 2012, *J Hum Evol* 63, 1-51) and corrected by equivalent measurements of A.L. 288-1 provided by those authors.
