# Supplementary material for: From Lucy to Kadanuumuu: balanced analyses of Australopithecus afarensis assemblages confirm only moderate skeletal dimorphism
Source: PeerJ. 2015 Apr 28;3:e925. doi: 10.7717/peerj.925 (PMC4419524; doi:10.7717/peerj.925)
Supplement: Table S2 [file peerj-03-925-s003.docx]

**Supplemental Table 2.** The Kadanuumuu (KSD-V/P-1/1) template sample used to calculate Geometric Mean Method dimorphism.

**Specimen CLAV HARB FLCL TMXT DSTB**

A.L. 128-1/129-1 - - 38.8 30.2 -

A.L. 137-48A - 36.7 - - -

A.L. 288-1 - 29.3 - 30.5 17.2

A.L. 322-1 - 34.6 - - -

A.L. 330-6 - - - 40.8 -

A.L. 333-4 - - 47.9 - -

A.L. 333-6 - - - - 22.4

A.L. 333-7 - - - - 25.8

A.L. 333-96 - - - - 23.1

A.L. 333w-56 - - 48.3 - -

A.L. 333x-6/9 12.0 - - - -

A.L. 333x-26 - - - 41.3 -

A.L. 545-3 - - - - 19.2

KSD-V/P-1-1 17.2 47.0 54.8 49.3 26.8
