# Supplementary material for: From Lucy to Kadanuumuu: balanced analyses of Australopithecus afarensis assemblages confirm only moderate skeletal dimorphism
Source: PeerJ. 2015 Apr 28;3:e925. doi: 10.7717/peerj.925 (PMC4419524; doi:10.7717/peerj.925)
Supplement: Table S3 [file peerj-03-925-s004.docx]

**Supplemental Table 3.** Descriptive statistics for individual metrics measured directly from chimpanzee, human and gorilla specimens.

**CLAV HHD HOCB HARB CAPD RHD ULB FHD FNKH TRCD GSTB FLCL CNDC PXTB TMXT DSTB FIBD TAL**

**Gorillas**

N 46 49 50 48 50 49 50 50 50 50 50 49 50 50 49 50 48 43

Mean 15.88 56.56 59.85 60.33 29.14 30.79 24.90 45.23 30.60 38.40 32.03 49.96 44.16 80.33 47.72 29.25 32.62 59.98

CV 15.28 14.91 18.09 14.30 15.02 14.59 16.74 12.65 14.01 12.67 14.97 14.03 14.22 13.62 16.33 13.80 16.06 10.98

Male N 23 24 25 23 25 24 25 25 25 25 25 24 25 25 24 25 23 21

Male Mean 17.76 64.44 69.61 68.50 32.94 34.85 28.38 50.41 34.10 42.29 36.18 56.39 49.50 90.15 54.94 32.59 37.47 65.77

Male CV 10.49 6.14 7.75 6.35 6.97 7.19 10.26 5.65 8.33 7.37 7.08 6.34 8.13 6.35 5.98 7.78 6.57 5.07

Female N 23 25 25 25 25 25 25 25 25 25 25 25 25 25 25 25 25 22

Female Mean 13.99 48.99 50.09 52.81 25.34 26.88 21.43 40.05 27.09 34.51 27.88 43.79 38.82 70.51 40.79 25.92 28.17 54.45

Female CV 7.51 4.31 6.37 4.01 7.43 4.82 6.02 3.93 7.14 7.64 7.42 4.79 5.47 4.25 7.03 7.23 7.64 5.86

Dimorphism 1.269 1.315 1.390 1.297 1.300 1.297 1.324 1.259 1.258 1.225 1.297 1.288 1.275 1.279 1.347 1.257 1.330 1.208

**Humans**

N 49 50 50 50 50 50 50 50 50 50 50 50 49 50 50 50 50 48

Mean 13.26 44.57 43.27 44.36 21.01 22.88 20.19 45.39 31.81 33.07 40.44 63.62 39.30 73.67 45.81 28.69 30.62 58.29

CV 14.52 9.04 11.00 8.92 9.24 9.12 13.80 8.95 11.00 9.41 9.14 6.23 10.22 7.89 10.77 11.81 9.60 8.10

Male N 25 25 25 25 25 25 25 25 25 25 25 25 25 25 25 25 25 24

Male Mean 14.55 48.13 46.62 47.45 22.24 24.54 22.17 48.67 34.39 35.15 42.56 66.37 42.38 78.45 48.53 30.79 32.27 61.99

Male CV 12.07 4.19 6.69 4.52 6.39 4.88 9.67 5.78 8.53 8.20 7.43 4.77 5.77 4.42 8.87 9.13 8.50 4.93

Female N 24 25 25 25 25 25 25 25 25 25 25 25 24 25 25 25 25 24

Female Mean 11.92 41.00 39.91 41.27 19.78 21.22 18.20 42.11 29.24 31.00 38.31 60.87 36.08 68.90 43.09 26.58 28.98 54.59

Female CV 7.70 3.81 9.05 6.59 8.04 6.08 9.53 4.25 5.48 5.14 7.65 4.10 6.89 4.41 9.26 9.49 7.36 5.04

Dimorphism 1.221 1.174 1.168 1.150 1.124 1.157 1.218 1.156 1.176 1.134 1.111 1.090 1.175 1.139 1.126 1.158 1.114 1.135

**Chimpanzees**

N 45 48 48 48 48 47 48 48 48 48 48 48 48 48 48 48 47 43

Mean 12.57 41.27 42.53 45.90 21.99 25.66 19.49 34.00 22.76 27.52 23.85 37.55 31.96 59.38 35.42 22.99 28.72 46.95

CV 8.40 6.64 9.42 6.35 9.49 6.64 9.03 6.47 7.28 7.80 7.32 6.79 7.41 5.98 7.65 8.20 6.74 6.23

Male N 21 23 23 23 23 22 23 23 23 23 23 23 23 23 23 23 22 19

Male Mean 12.96 42.65 44.10 47.17 22.97 26.17 20.07 34.84 23.13 28.27 24.21 38.48 32.55 60.63 36.17 23.03 29.50 47.71

Male CV 6.14 4.78 8.24 4.12 7.90 4.38 7.01 4.43 6.71 7.50 7.73 6.81 6.55 4.86 5.17 7.80 5.44 4.82

Female N 24 25 25 25 25 25 25 25 25 25 25 25 25 25 25 25 25 24

Female Mean 12.23 40.00 41.09 44.74 21.08 25.22 18.95 33.23 22.41 26.82 23.53 36.69 31.42 58.23 34.73 22.96 28.04 46.35

Female CV 9.46 6.83 9.44 7.17 9.23 7.95 10.11 7.41 7.67 7.37 6.85 6.04 7.96 6.43 9.23 8.78 7.08 7.09

Dimorphism 1.060 1.066 1.073 1.055 1.089 1.038 1.059 1.049 1.032 1.054 1.029 1.049 1.036 1.041 1.041 1.003 1.052 1.029
