# Supplementary material for: From Lucy to Kadanuumuu: balanced analyses of Australopithecus afarensis assemblages confirm only moderate skeletal dimorphism
Source: PeerJ. 2015 Apr 28;3:e925. doi: 10.7717/peerj.925 (PMC4419524; doi:10.7717/peerj.925)
Supplement: Table S4 [file peerj-03-925-s005.docx]

**Supplemental Table 4.** Results of simulations modeling only 5 mixed sex individuals preserved at A.L. 333.

**Dimorphism**

**Simulation Value Chimp Human Gorilla**

Lucy Template – CA (N=42) Mean (sd) < > Mean (sd) < > Mean (sd) < >

BDI 1.207 1.161 (0.026) 965 45 1.203 (0.031) 612 388 1.286 (0.041) 23 977

CV 11.75 9.48 (1.39) 946 54 11.57 (1.574) 573 427 15.28 (1.72) 17 983

Geometric Mean Method Mean (sd) < > Mean (sd) < > Mean (sd) < >

Full Lucy Sample 1.299 1.117 (0.018) 1000 0 1.158 (0.026) 1000 0 1.244 (0.043) 897 103

130% Lucy 1.179 1.117 (0.018) 998 2 1.158 (0.026) 794 206 1.244 (0.043) 54 946

Single Lucy & 128/129 1.176 1.115 (0.019) 998 2 1.154 (0.026) 810 190 1.234 (0.043) 73 937
